# Body mass index and risk of non-melanoma skin cancer: cumulative evidence from prospective studies

**DOI:** 10.1038/srep37691

**Published:** 2016-11-29

**Authors:** Daijun Zhou, Jun Wu, Gaoxing Luo

**Affiliations:** 1 Institute of Burn Research; State Key Laboratory of Trauma, Burn and Combined Injury; Key Laboratory of Proteomics of Chongqing, Southwest Hospital, Third Military Medical University, Chongqing 400038, China

## Abstract

Prospective epidemiologic studies that investigated the association between body mass index (BMI) and non-melanoma skin cancer (NMSC) yielded inconsistent findings. A dose-response meta-analysis was conducted to quantitatively summarize the evidence. PubMed and Embase databases were searched for relevant studies. Study-specific relative risk (RR) and 95% confidence interval (CI) for an increase in BMI of 5 kg/m^2^ was computed with the generalized least squares trend estimation, and these risk estimates were combined with the random-effects model. Nine publications were included in the final analyses, consisting of 18 independent cohorts with 22 risk estimates (971,795 participants and 50,561 NMSC cases). Results of the dose-response analyses showed a nonlinear inverse relationship between BMI and NMSC (RR = 0.88, 95% CI: 0.85–0.91, *I*^2^ = 71.2%, *P*-nonlinearity <0.001), which persisted when limiting to the studies with adjustment for important potential confounders including sun exposure and sensitivity factors. The risk estimates were very similar for squamous cell carcinoma and basal cell carcinoma. Sex appeared a source of heterogeneity (*P*-difference = 0.06), with a weaker, but still significant inverse association in men than in women. This dose-response meta-analysis suggests a nonlinear inverse association between BMI and NMSC.

Non-melanoma skin cancer (NMSC), generally referring to squamous cell carcinoma (SCC) and basal cell carcinoma (BCC), ranks the most frequently diagnosed cancer worldwide. Unlike other cancers for which the incidence rates has become stabilized or declined, the incidence of NMSC is still increasing, in particular among younger populations^1^,^2^. Although the mortality rate of NMSC is much lower than that of melanoma, the high incidence lead to a considerable number of cases, and therefore an important challenge in health management and enormous burden in terms of healthcare costs^3^. In the US alone, it is estimated that in 2012 there were more than 5.4 million NMSCs and 3.3 million persons treated for NMSC^2^. More than 80% of NMSC incidence could be attributed to ambient ultraviolet (UV) radiation^4^, and several host features such as fair skin, red hair, melanocytic nevi, and family history have also been identified as risk factors^5^,^6^. The effectiveness of skin cancer screening on reducing mortality is suggested to be low^7^. Thus, identifying other exposures, especially modifiable factors (e.g., diet and lifestyle) that influence the risk for this malignancy is important for reducing the public health burden.

Obesity, as measured by body mass index (BMI), is an established risk factor for various chronic diseases such as type 2 diabetes, cardiovascular disease, and cancers at various sites, as well as all-cause mortality^8^. Regarding the association with skin cancer, previous meta-analyses reported increased risk of melanoma associated with a higher BMI in men but not in women^5^,^9^. Given that melanoma and NMSC share risk factors^5^, it is plausible that excess body weight would also be associated with NMSC risk. There have been a number of prospective observational studies that investigated this association, but their findings are inconsistent^10^,^11^,^12^,^13^,^14^,^15^,^16^,^17^,^18^. Indeed, some^10^,^11^,^16^,^18^, but not other studies^12^,^13^,^14^,^15^ have demonstrated an unexpectedly inverse association between measured/self-reported BMI and NMSC risk. The reasons for the heterogeneity among these studies are unclear, and whether there is a dose-response association, or whether such an inverse association is simply explained by confounders of sun exposure and sensitivity measures remain to be elucidated (obese individuals have less sun exposure than lean ones because of a lower frequency of outdoors^18^). Therefore, we performed a dose-response meta-analysis of prospective studies to investigate the relationship between BMI and risk of NMSC.

## Methods

### Literature search

Potentially relevant publications were searched on PubMed and Embase databases from inception to 30 June, 2016. The detailed literature search strategy is reported in Supplementary Table S1, with core search involving NMSC and its subtype (SCC and BCC), anthropometric factors, and study design. The references of retrieved full publications were also carefully hand searched for additional studies.

### Study selection

Studies were eligible for inclusion if they met the following criteria: (1) the study design was prospective (e.g., prospective cohort, nested case-control, or case-cohort design); (2) the exposure of interest was BMI (in kg/m^2^), or obesity status measured by BMI; (3) the outcome of interest was NMSC or its subtype; and (4) relative risks (RRs) and 95% confidence intervals (CIs) were reported for continuous BMI (e.g., per unit increase), or for at least 3 quantitative categories of BMI. When the same study was reported in overlapping publications, the one containing the largest number of NMSCs was included.

### Data extraction and quality assessment

For study included, the following characteristics were collected a standardized data-collection form: the first author’s last name, publication year, study name, country, average (mean or median) duration of follow-up, sex and age of participants, number of cases and participants, methods for BMI measurement, categories of BMI with corresponding RR and 95% CI, and potential confounders accounted for in the statistical analyses. Two authors (ZD and LG) independently conducted literature search, study selection, and data abstraction, with any disagreement resolved by consensus. Instead of assessing methodologic quality of the primary studies by a quality score, we investigated whether the summary risk estimates were significantly influenced by some study and population characteristics (e.g., follow-up duration, method for exposure assessment, and adjustment for important confounders) which may be indicators of study quality^19^.

### Statistical analysis

In this meta-analysis, we used the most fully adjusted risk estimates, and reported summary RR of NMSC for each 5 kg/m^2^ increase in BMI. When only results for categorized BMI were reported, the method of generalized least squares trend estimation proposed by Greenland and Longnecker^20^ and Orsini *et al.*^21^ was employed to estimate study-specific slopes and 95% CIs from the correlated logs of the RRs and CIs across BMI categories. Accordingly, categorized BMI values, distributions of cases and person-years, and RRs with 95% CIs were extracted from each study. When the number of cases or person-years in each exposure category was not reported, the data were estimated from total number of cases or person-years. The median or mean BMI in each category were used as the average levels. When the median or mean value per category was not reported, the midpoint of the upper and lower boundaries was considered average values. If the highest or lowest category was open-ended, the width of the interval was assumed to be the same as in the closest category. Afterward, study-specific risk estimates were combined with a random-effects model, taking into account both within- and between-study variation^22^. Results presented by sex or by NMSC subtype in original studies were treated as independent reports.

To explore potential sources of heterogeneity, subgroup analyses were performed by geographic area, sex of participants, NMSC subtype (SCC and BCC), methods for BMI measurement, and adjustment for important confounders. Further sex-specific analyses were conducted for SCC and BCC. A potential nonlinear association between BMI and risk of NMSC were investigated using restricted cubic splines with three knots at percentiles 10%, 50%, and 90% of the distribution^23^. A *P* value for non-linearity was calculated by testing the null hypothesis that the coefficient of the second spline is equal to 0. Heterogeneity among studies was assessed with the *Q* and *I*^2^ statistics^24^. For the *Q* statistic, a *P* < 0.1 was considered significant heterogeneity. Potential publication bias was assessed by a combination of Begg rank correlation test, Egger linear regression test, and Begg’s funnel plot^25^,^26^. All analyses were performed using STATA12.0.

## Results

### Study selection and characteristics

The flow diagram of study selection is reported in Supplementary Figure S1. Our initial search yielded 1215 independent citations, of which 32 full publications remained after title and/or abstract reading. Twenty three of these full publications were rejected mainly because of a retrospective study design, or a lack of results reported for BMI and/or NMSC. Of note, 1 report^27^ was excluded because only results for total skin cancer were available, and another report^28^ was excluded because the same study population was used in a later publication^16^ with larger events.

Finally, 9 publications^10^,^11^,^12^,^13^,^14^,^15^,^16^,^17^,^18^ that evaluated the association of BMI with risk of NMSC were included in this meta-analysis, consisting of 1 nested case-control study^13^ of sex-same twins, 1 pooled analysis^14^ of 7 cohorts from 3 European countries, 1 pooled analysis^10^ of 2 cohorts (the Copenhagen General Population Study and the Copenhagen City Heart Study), and 7 independent prospective cohort studies^11^,^12^,^15^,^16^,^17^,^18^ (the Health Professionals Follow-up Study and the Nurses’ Health Study were reported in 1 publication^16^). Therefore, a total of 18 cohorts were included in this meta-analysis, consisting of 22 independent reports with 971,795 participants and 50,561 NMSC cases (3332 SCC and 33,721 BCC). The primary studies were mostly from Europe^10^,^13^,^14^,^17^ or US^11^,^16^,^18^, with the exceptions of 2 Australia studies^12^,^15^. BMI was either self-reported, or measured by investigators. The study duration, sample size, and potential confounders adjusted for varied substantially among individual studies. Three of the original studies were observational analyses of clinical trials on cancer prevention (skin cancer^12^,^15^ or female breast cancer^18^). The characteristics of the included studies are reported in Supplementary Table S2.

### Overall analyses

**Figure 1:**
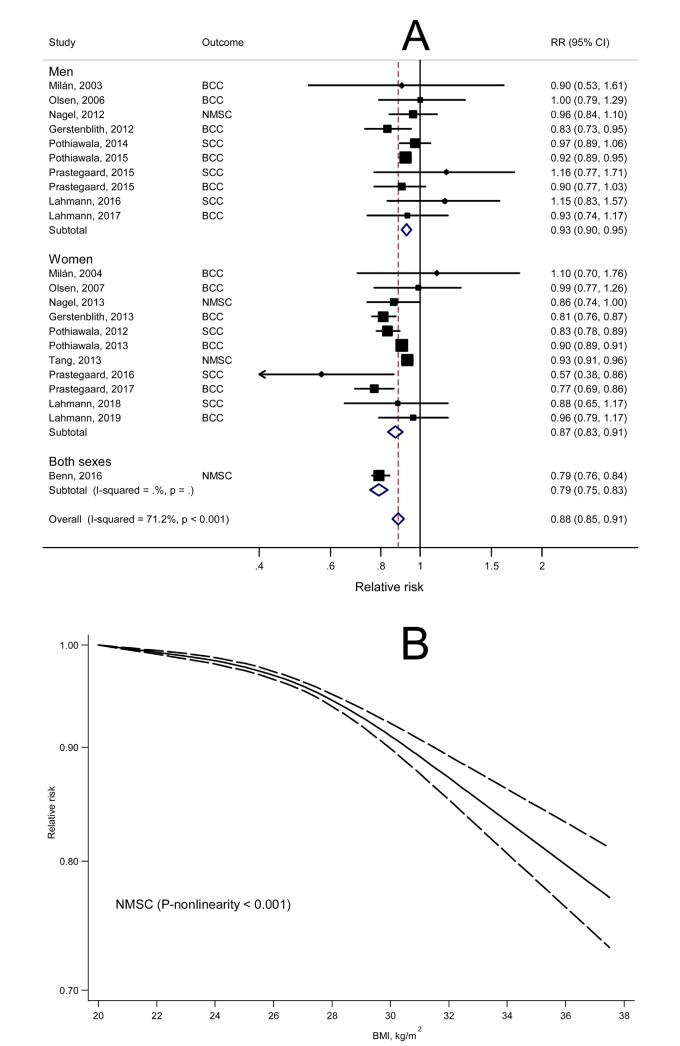
Dose-response meta-analyses of body mass index (BMI) and risk of non-melanoma skin cancer (NMSC). A, linear dose-response analyses for BMI increase of 5 kg/m2; B, nonlinear dose-response analyses.

A dose-response meta-analysis of the included studies showed that the summary RR of NMSC was 0.88 (95% CI: 0.85–0.91) for each 5 kg/m^2^ increase in BMI, with considerable heterogeneity (*P*_heterogeneity_ < 0.001, *I*^2^ = 71.2%) (Fig. 1A). There was evidence of a nonlinear relationship between BMI and risk of NMSC, with the curve becoming steeper and almost linear after the BMI of 28 kg/m^2^ (Fig. 1B). Both Begg rank correlation test and Egger linear regression test suggested little evidence of publication bias (both *P* values > 0.50), which was supported by a visual inspection of the Begg funnel plot that showed no obvious asymmetry (Supplementary Figure S2).

### Subgroup and sensitivity analyses

**Figure 2:**
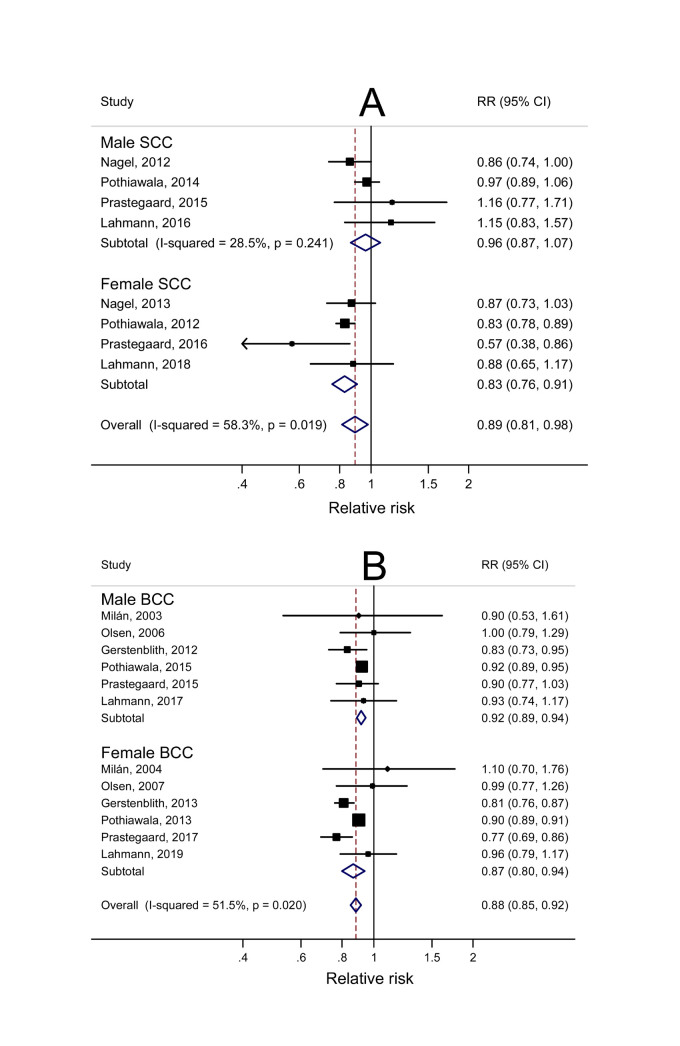
Overall and sex-specific dose-response meta-analyses of body mass index (per 5 kg/m2 increase) and risk of squamous cell carcinoma (SCC) and basal cell carcinoma (BCC).

**Figure 3:**
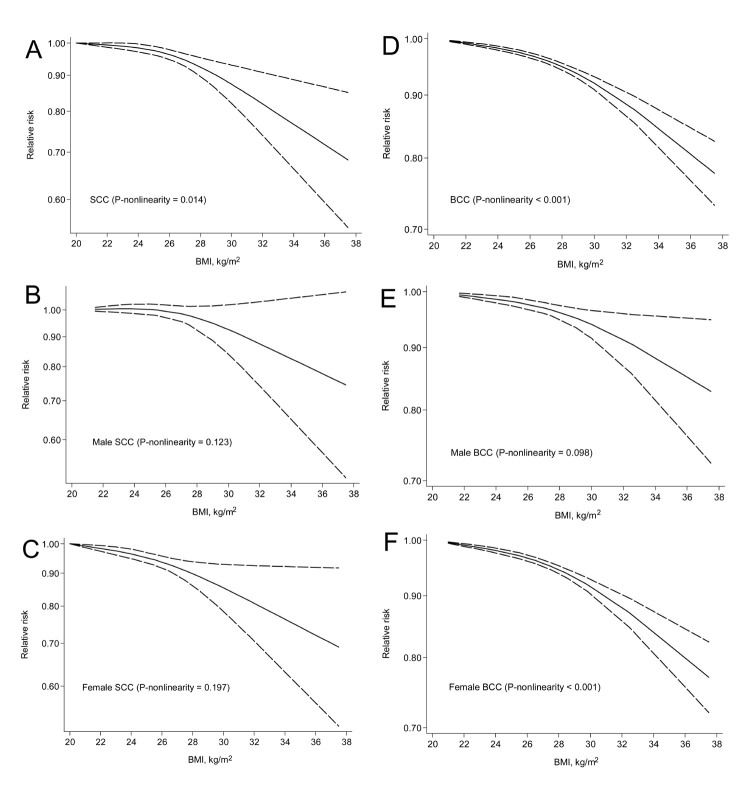
Overall and sex-specific, nonlinear dose-response meta-analyses of body mass index (BMI) and risk of squamous cell carcinoma (SCC) and basal cell carcinoma (BCC).

Results of subgroup analysis performed to explore potential sources of heterogeneity according to pre-specified factors are reported in Table 1. The magnitude of the association was very similar for SCC and BCC. There was evidence that geographic area partly contributed to the heterogeneity amongst studies, with an inverse BMI-NMSC association among US and European studies, but not among studies from Australia (*P*_difference_ = 0.05 when comparing European and Australia studies). There also a suggestion of an effect modification by sex (*P*_difference_ = 0.06), with a weaker but still significant association in men than in women (Fig. 1A and Table 1). This sex-specific difference appeared more evident for SCC than for BCC (Fig. 2, Fig. 3 and Table 1). There was evidence that the association varied by whether adjustment for alcohol consumption had been made (*P*_difference_ = 0.004), but only 3 reports were included in the group with adjustment. Of note, the association did not differ according to adjustment or not for several well known risk factors for NMSC such as family history and indicators of sun exposure and sun sensitivity. Excluding the 3 studies (7 reports) where participants were generated from clinical prevention trials, the summary RR was 0.86 (95% CI: 0.83–0.90), with substantial heterogeneity (*I*^2^ = 77.0%).

## Discussion

In this dose-response meta-analysis of prospective studies involving 18 independent cohorts with nearly 1 million participants and over 50,000 NMSC cases, we found that BMI was nonlinearly, inversely associated with risk of NMSC. The association was similar for SCC and BCC, and persisted when limiting to the studies with adjustment for important potential confounders including sun exposure and sun sensitivity measures. We also found that the inverse association in women was more evident than in men.

Potential biological mechanisms under the observed inverse association between BMI and NMSC risk are poorly understood. Some investigators attributed this association to the residual confounding by sun exposure^16^,^17^,^18^. Obese compared with lean individuals are less active outdoor, and therefore less sun exposure^18^, leading to a lower risk of NMSC. However, our meta-regression analyses suggested that neither adjustment for physical activity, nor adjustment for sun exposure and sensitivity significantly influenced the magnitude of the association. Moreover, UV radiation appears to affect SCC more strongly than on BCC^29^,^30^,^31^. If the inverse BMI-NMSC association could be simply explained by residual confounder of sun exposure, per unit increase in BMI would be expected to be associated with greater reductions in SCC than in BCC. However, risks of SCC and BCC associated with increasing BMI were very similar in this meta-analysis. Using a Mendelian randomization design is an approach to exclude potential confounders. There is only 1 prospective study that used such a design, showing that an increase in genetically determined BMI of 10 kg/m^2^ was non-significantly inversely associated with NMSC risk (RR = 0.71, 95% CI: 0.26–1.93)^10^.

Gerstenblith *et al.*^11^ argued that adipose tissue related high estrogen may partly be responsible for the inverse association of BMI with NMSC risk. We considered, but not limited to the following evidence that supports this argument: (1) women relative to men have much lower risk of NMSC^32^, suggesting that women may have some protections; (2) animal studies showed that withdrawal of ovary dramatically increased susceptibility to NMSC in female mice^33^, and estrogen receptor-β agonist remarkably decreased UV-induced skin cancer^34^; and (3) There is evidence that estrogen may promote wound healing and delay or even reverse skin aging through multiple mechanisms (e.g., reduction of inflammation and oxidative damage)^35^,^36^. Another hormone that is significantly higher in women than in men is adipokine, leptin, which has also been suggested to promote wound repair in the skin (e.g., by accelerating proliferation and enhancing angiogenesis around the wounded)^37^, and to improve, preserve and restore skin regeneration^38^. For men and women having an equivalent amount of excess weight, women carry more subcutaneous fat which a major producer of leptin, and this might partly explain the sex-specific difference in the association of BMI with NMSC risk in this study.

Evidence from case-control studies on BMI and NMSC has been limited, and some also suggested a sex-specific difference. In a small, hospital-based case-control study of 77 NMSC^39^, being obese was inversely associated with BCC (odds ratios [OR] = 0.53, 95% CI: 0.05–5.24), but was positively associated with total NMSC (OR = 1.51, 95% CI: 0.27–8.50). In another hospital-based study of 528 BCC^40^, women in the fourth quartile of BMI had a 34% (OR = 0.66, 95% CI: 0.35–1.22) non-significantly lower risk of BCC as compared with women in the first quartile, whereas the corresponding OR for men was 0.97 (95% CI: 0.58–1.63). In a population-based case-control study of 1276 NMSC^41^, obesity was associated with a non-significant increase in risk of NMSC in men (OR = 1.25, 95% CI: 0.94–1.64); conversely, a trend towards reduced risk was found in women (OR = 0.79, 95% CI: 0.62–1.01).

This meta-analysis presents several strengths. Limiting our analyses to prospective studies eliminated reverse causality because BMI was determined before the diagnosis of cancer. The large number of cases strengthened the statistical power to detect a weak association. Our linear and nonlinear dose-response analyses enabled us to better understand the shape of the association. Moreover, the stability of our results was supported by the stratified analyses according to various important study characteristics. Heterogeneity and publication bias are two important concerns for a meta-analysis of published literature, because different studies would vary by population and methodological characteristics, and small studies with null results have less chance to be published. However, with commonly used statistical methods, we found little evidence of publication bias. We were also able to identify potential sources of heterogeneity by performing meta-regression analyses.

However, this study is not without limitations. As a meta-analysis of observational studies, the problem of potential confounders is the first consideration. Although the summary risk estimates were not altered when restricting to the studies with sun exposure and sensitivity adjustment, residual confounding arising from measurement errors of sun exposure (and also other factors) remained. Original studies generally used self-reported questionnaires to collect lifestyle information, and the way in which they asked about the duration and density of the exposure varied across different questionnaires; moreover, other problems such as the use of sunscreen and the degree to which clothing covers the body surface were less or not considered in these cohorts. Therefore, future prospective studies with more accurate measures of factors related to both BMI and sun exposure, and those studies using a Mendelian randomization design are needed to further address the impacts of these confounders on the association.

In summary, findings from this meta-analysis of prospective studies suggest that BMI is nonlinearly and inversely association with risk of NMSC. The inverse association is similar with SCC and BCC, but it appears stronger in women than in men. Potential mechanism underlying the association remains to be determined. Despite that we and others have observed lower risks of specific cancers (e.g., NMSC and lung cancer^42^) associated with a higher BMI, these findings should not affect current preventive strategies on obesity, which is an important cause of serious public health concerns worldwide^8^.

## Additional Information

**How to cite this article**: Zhou, D. *et al.* Body mass index and risk of non-melanoma skin cancer: cumulative evidence from prospective studies. *Sci. Rep.* **6**, 37691; doi: 10.1038/srep37691 (2016).

**Publisher's note:** Springer Nature remains neutral with regard to jurisdictional claims in published maps and institutional affiliations.

## Supplementary Material

Supplementary Information

## Figures and Tables

**Table 1:** Subgroup analysis for the dose-response relationship between BMI and NMSC.

	No. of reports	RR (95% CI)	*P* for heterogeneity	*I*^2^ (%)	*P* for difference
Geographic area					
US	7	0.89 (0.86–0.92)	<0.001	77.4	Ref
Europe	9	0.85 (0.78–0.92)	0.02	55.9	0.32
Australia	6	0.97 (0.88–1.08)	0.88	0	0.15, 0.05^a^
Sex of participants					
Men	10	0.93 (0.90–0.95)	0.57	0	
Women	11	0.87 (0.83–0.91)	<0.001	71.8	0.06
NMSC subtype					
SCC	8	0.89 (0.81–0.98)	0.02	58.3	
BCC	12	0.88 (0.85–0.92)	0.02	51.5	0.85
Male SCC	4	0.96 (0.87–1.07)	0.24	28.5	
Female SCC	4	0.83 (0.76–0.91)	0.30	18.8	0.04
Male BCC	6	0.92 (0.89–0.94)	0.73	0	
Female BCC	6	0.87 (0.80–0.94)	0.003	72.7	0.36
Average duration of follow-up					
≥10 years	16	0.90 (0.87–0.93)	0.02	48.6	
<10 years	6	0.87 (0.85–0.97)	<0.001	88.2	0.34
Assessment of BMI					
Measured	13	0.89 (0.82–0.96)	0.004	58.2	
Self-reported	9	0.89 (0.87–0.92)	0.001	70.7	0.77
Statistical adjustment					
Smoking					
Yes	10	0.88 (0.82–0.94)	<0.001	80.1	
No	12	0.89 (0.86–0.93)	0.006	58.4	0.66
Alcohol consumption					
Yes	3	0.80 (0.77–0.83)	0.71	0	
No	19	0.91 (0.88–0.93)	0.009	48.9	0.004
Physical activity					
Yes	7	0.86 (0.82–0.91)	<0.001	86.8	
No	15	0.91 (0.86–0.96)	0.08	36.3	0.22
History of NMSC					
Yes^b^	13	0.90 (0.87–0.93)	0.002	60.7	0.13
No	9	0.85 (0.78–0.92)	0.02	55.9	
Family history of NMSC					
Yes	6	0.90 (0.87–0.93)	0.05	54.6	
No	16	0.88 (0.83–0.93)	<0.001	74.8	0.41
Both sun exposure and sensitivity					
Yes	7	0.89 (0.86–0.92)	<0.001	77.4	
No	15	0.89 (0.83–0.96)	0.007	53.6	0.91

BCC, basal cell carcinoma; BMI, body mass index; NMSC, non-melanoma skin cancer; SCC, squamous cell carcinoma.

^a^Australian studies compared with European studies.

^b^Either excluding NMSC at baseline or adjusting for the presence of NMSC.
