# Supplementary material for: Body mass index and risk of non-melanoma skin cancer: cumulative evidence from prospective studies
Source: Sci Rep. 2016 Nov 29;6:37691. doi: 10.1038/srep37691 (PMC5127294; doi:10.1038/srep37691)
Supplement: Supplementary Information [file srep37691-s1.pdf]

Daijun Zhou, Jun Wu, and Gaoxing Luo. Body mass index and risk of non-melanoma skin cancer: cumulative evidence from prospective studies.

Supplementary Table S1. Characteristics of included prospective studies on the association of body mass index with risk of NMSC.

Study	Source of population	Country	Average duration	Sex and baseline age[a]	No. of cases/ Participants	Measurement of BMI	BMI categories, kg/m^2	RR (95% CI)	Variables adjustment
Milán, 2003 (nested case-control)[1]	Finnish Adult Twin Cohort Study	Finland	15.2 yr	M&W	M: 149 BCC/298<br>W: 184 BCC/368	Self-reported	Per 1 unit	M:<br>0.98 (0.88-1.00)<br>W:<br>1.02 (0.93-1.12)	Sex-same tiwn pairs.
Olsen, 2006[2]	Nambour Skin Cancer Prevention Trial	Australia	4.5 yr	M: 50.7 yr<br>W: 49.6 yr	M: 66 BCC/449<br>W: 75 BCC/511	Measured	M&W<br><25<br>25-29.9<br>≥30	M:<br>1.0<br>0.9 (0.6-1.7)<br>1.0 (0.6-1.7)<br>W:<br>1.0<br>0.7 (0.5-1.1)<br>1.2 (0.7-2.0)	Age and history of BCC.
Nagel, 2012[3]	Metabolic Syndrome and Cancer Project	Austria, Norway, and Sweden	12.2 yr	M: 43.9 yr<br>W: 44.1 yr	M: 786 NMSC, 587 SCC /289,886<br>W: 396 NMSC, 286 SCC/288,834	Measured	M (mean BMI):<br>21.5<br>23.8<br>25.4<br>27.1<br>30.8<br><br><br><br><br><br><br>W (mean BMI):<br>20.0<br>22.3<br>24.1<br>26.4<br>31.7	M (NMSC):<br>1.00<br>1.21 (0.93-1.58)<br>1.22 (0.94-1.58)<br>1.05 (0.81-1.37)<br>1.00 (0.77-1.31)<br>M (SCC):<br>1.00<br>1.12 (0.84-1.51)<br>1.17 (0.87-1.56)<br>0.97 (0.72-1.30)<br>0.78 (0.57-1.06)<br>W (NMSC):<br>1.00<br>0.92 (0.62-1.36)<br>0.62 (0.42-0.93)<br>0.84 (0.58-1.21)<br>0.69 (0.48-1.01)<br>W (SCC):<br>1.00<br>0.90 (0.57-1.42)<br>0.65 (0.41-1.03)<br>0.79 (0.51-1.21)<br>0.71 (0.46-1.10)	Age, smoking, study center, year of birth, and measurement error (corrected by regression dilution ratio).

Gerstenblith, 2012[4]	United States Radiological Technologists cohort	US	8.75 yr	M: 48.7-49.8 yr in different quartiles<br><br>W: 46.2-48.3 yr in different quartiles	M: 480 BCC/11,631<br>W: 1768 BCC/46,587	Self-reported	M&W:<br><25<br>25-<30<br>30-<35<br>≥ 35	M:<br>1.00<br>0.86 (0.70-1.05)<br>0.64 (0.47-0.87)<br>0.65 (0.39-1.08)<br>W:<br>1.00<br>0.74 (0.66-0.83)<br>0.67 (0.56-0.81)<br>0.57 (0.44-0.77)	Age, education, income, smoking, alcohol intake, physical activity, hair, eye, and skin color, geographic measure of sun exposure (TOMS) from five age periods, hours outdoors in summer in five age periods, number of lifetime blistering sunburns, acute and chronic reactions to sunlight, cumulative occupational ionizing radiation dose from head/neck.
Pothiawala, 2012[5]	Health Professionals Follow-up Study (HPFS);<br>Nurses' Health Study (NHS)	US	16.0 yr (HPFS), 26.3 yr (NHS)	M (age range): 30-55 yr<br>W (age range): 40-75 yr	1930 SCC, 27200 BCC/ 40381 M , 102748 W	Self-reported	M&W:<br>18.5-24.9<br>25-29.9<br>30-34.9<br>≥ 35	M (SCC):<br>1.00<br>1.00 (0.86-1.16)<br>0.82 (0.62-1.08)<br>1.07 (0.64-1.78)<br>M (BCC):<br>1.00<br>0.89 (0.85-0.95)<br>0.83 (0.76-0.90)<br>0.83 (0.70-0.98)<br>W (SCC):<br>1.00<br>0.80 (0.70-0.91)<br>0.65 (0.53-0.80)<br>0.59 (0.43-0.80)<br>W (BCC):<br>1.00<br>0.88 (0.85-0.91)<br>0.81 (0.78-0.85)<br>0.70 (0.65-0.75)	Age, physical activity, family history of melanoma, sunburn reaction, number of severe sunburns, number of moles, hair color, sun exposure at different age intervals, UV index at residence at different ages, and history of cardiovascular disease, type 2 diabetes, and cancer.
Tang, 2013[6]	Women's Health Initiative Observational Study	US	9.4	W (age range): 50-79 yr	9870 NMSC /61,657	Self-report	18.5-24.9<br>25.0-29.9<br>≥30.0	1.00<br>0.93 (0.89-0.99)<br>0.86 (0.80-0.91)	Age, education, smoking, skin type, sun exposure, history of skin cancer, use of hormone replacement therapy, and sunscreen use.
Præstegaard, 2015[7]	Danish Diet, Cancer, and Health Cohort study	Denmark	14.4	M&W (age range): 50-65 yr	M: 203 SCC, 1671 BCC/26685<br>W: 138 SCC, 1794 BCC/29243	Measured	M:<br>≤24<br>>24-26<br>>26-28<br>>28<br>Per 2 unit	M (SCC):<br>1.00<br>0.93 (0.62-1.39)<br>0.78 (0.48-1.26)<br>0.87 (0.49-1.58)<br>1.06 (0.90-1.24)<br>M (BCC):<br>1.00<br>1.07 (0.93-1.23)<br>1.00 (0.85-1.18)	Age, waist circumference, sun sensitivity, degree of freckling, number of nevi.

							 <br>W:<br>≤22<br>>22-24<br>>24-27<br>>27<br>Per 2 unit	0.85 (0.69-1.05)<br>0.96 (0.90-1.01)<br>W (SCC):<br>1.00<br>0.91 (0.56-1.50)<br>0.68 (0.39-1.16)<br>0.46 (0.22-0.97)<br>0.80 (0.68-0.94)<br>W (BCC):<br>1.00<br>0.88 (0.76-1.01)<br>0.83 (0.72-0.97)<br>0.67 (0.54-0.82)<br>0.90 (0.86-0.94)	
Lahmann, 2016[8]	Nambour Skin Cancer Prevention Trial	Australia	14.4	M: 44.3-52.2 yr in different quartiles<br>W: 49.4-51.8 yr In different quartiles	M: 98 SCC, 160 BCC/506<br>W: 90 SCC, 174 BCC/665	Measured	M:<br>≤24.1<br>>24.1-26.3<br>>26.3-28.8<br>>28.8<br><br><br><br><br><br>W:<br>≤22.7<br>>22.7-24.9<br>>24.9-28.2<br>>28.2	M (SCC):<br>1.00<br>1.04 (0.66-1.65)<br>0.98 (0.59-1.62)<br>1.23 (0.77-1.95)<br>M (BCC):<br>1.00<br>0.84 (0.60-1.17)<br>0.79 (0.56-1.11)<br>0.91 (0.65-1.27)<br>W (SCC):<br>1.00<br>0.58 (0.35-0.99)<br>0.64 (0.37-1.05)<br>0.78 (0.47-1.27)<br>W (BCC):<br>1.00<br>0.96 (0.67-1.37)<br>0.90 (0.64-1.27)<br>0.95 (0.68-1.33)	Age, smoking, treatment allocation, history of BCC or SCC, elastosis of the neck, and freckling of the back.
Benn, 2016[9]	Copenhagen General Population Study; Copenhagen City Heart Study	Denmark	4.7	M&W: 56.3 yr	M&W<br>3347<br>NMSC/108,812	Measured	M&W<br>18.5-24.9<br>25.0-29.9<br>≥30.0<br>Per 10 unit	M&W<br>1.00<br>0.82 (0.76-0.89)<br>0.65 (0.58-0.72)<br>0.63 (0.58-0.70)	Age, sex, education, smoking, alcohol intake, physical activity, C-reactive protein concentrations, birth year, and menopausal status for W.

BCC, basal cell carcinoma; BMI, body mass index; M, men; NMSC, non-melanoma skin cancer; SCC, squamous cell carcinoma; W, women; yr, years

[a] Unless explicitly stated, the age reported here is a mean value.

References

1 Milan, T., Verkasalo, P. K., Kaprio, J. & Koskenvuo, M. Lifestyle differences in twin pairs discordant for basal cell carcinoma of the skin. *Br J Dermatol* **149**, 115-23 (2003).

2 Olsen, C. M., Hughes, M. C., Pandeya, N. & Green, A. C. Anthropometric measures in relation to basal cell carcinoma: a longitudinal study. *BMC Cancer* **6**, 82 (2006).

3 Nagel, G. *et al.* Metabolic risk factors and skin cancer in the Metabolic Syndrome and Cancer Project (Me-Can). *Br J Dermatol* **167**, 59-67 (2012).

4 Gerstenblith, M. R. *et al.* Basal cell carcinoma and anthropometric factors in the U.S. radiologic technologists cohort study. *Int J Cancer* **131**, E149-55 (2012).

5 Pothiawala, S., Qureshi, A. A., Li, Y. & Han, J. Obesity and the incidence of skin cancer in US Caucasians. *Cancer Causes Control* **23**, 717-26 (2012).

6 Tang, J. Y. *et al.* Lower skin cancer risk in women with higher body mass index: the women's health initiative observational study. *Cancer Epidemiol Biomarkers Prev* **22**, 2412-5 (2013).

7 Praestegaard, C. *et al.* Obesity and risks for malignant melanoma and non-melanoma skin cancer: results from a large Danish prospective cohort study. *J Invest Dermatol* **135**, 901-4 (2015).

8 Lahmann, P. H., Hughes, M. C., Williams, G. M. & Green, A. C. A prospective study of measured body size and height and risk of keratinocyte cancers and melanoma. *Cancer Epidemiol* **40**, 119-25 (2016).

9 Benn, M., Tybjaerg-Hansen, A., Smith, G. D. & Nordestgaard, B. G. High body mass index and cancer risk-a Mendelian randomisation study. *Eur J Epidemiol*(2016).

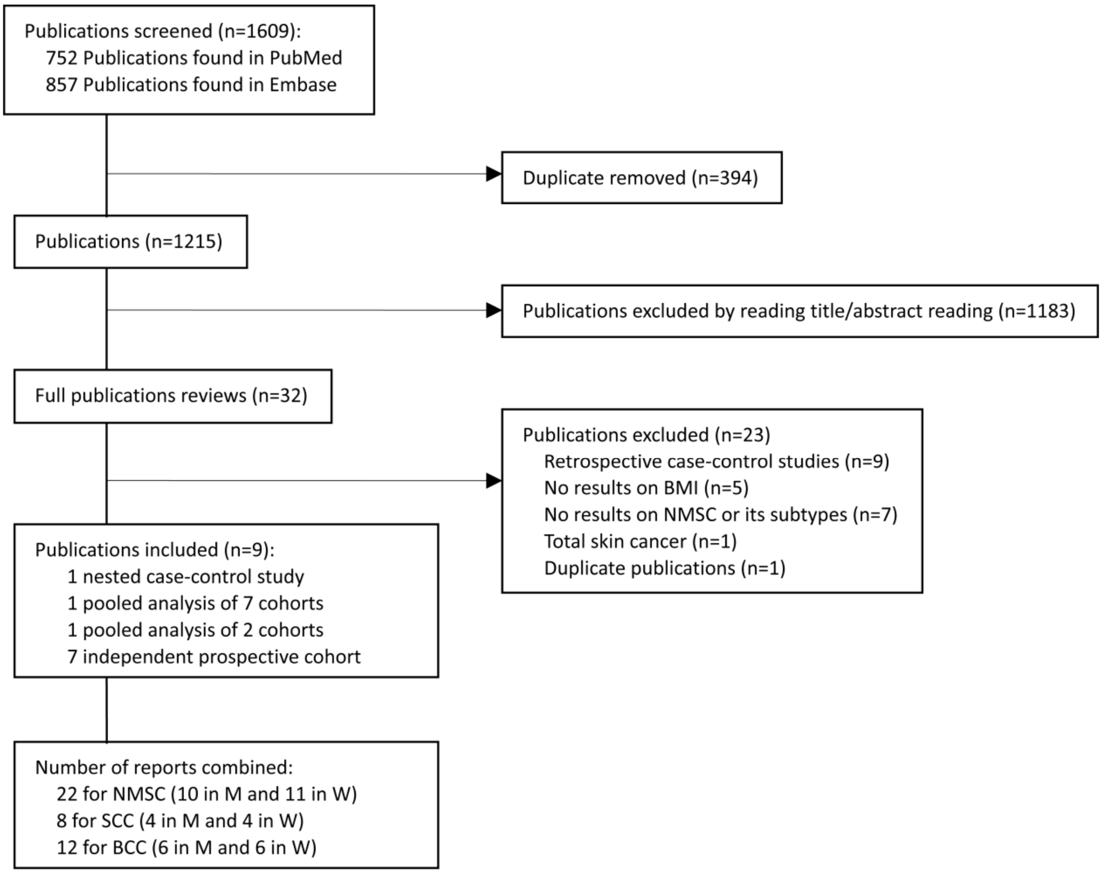

Supplementary Figure S1. The flow diagram of study selection for the meta-analysis. BCC, basal cell carcinoma; BMI, body mass index; M, men; NMSC, non-melanoma skin cancer; SCC, squamous cell carcinoma; W, women;

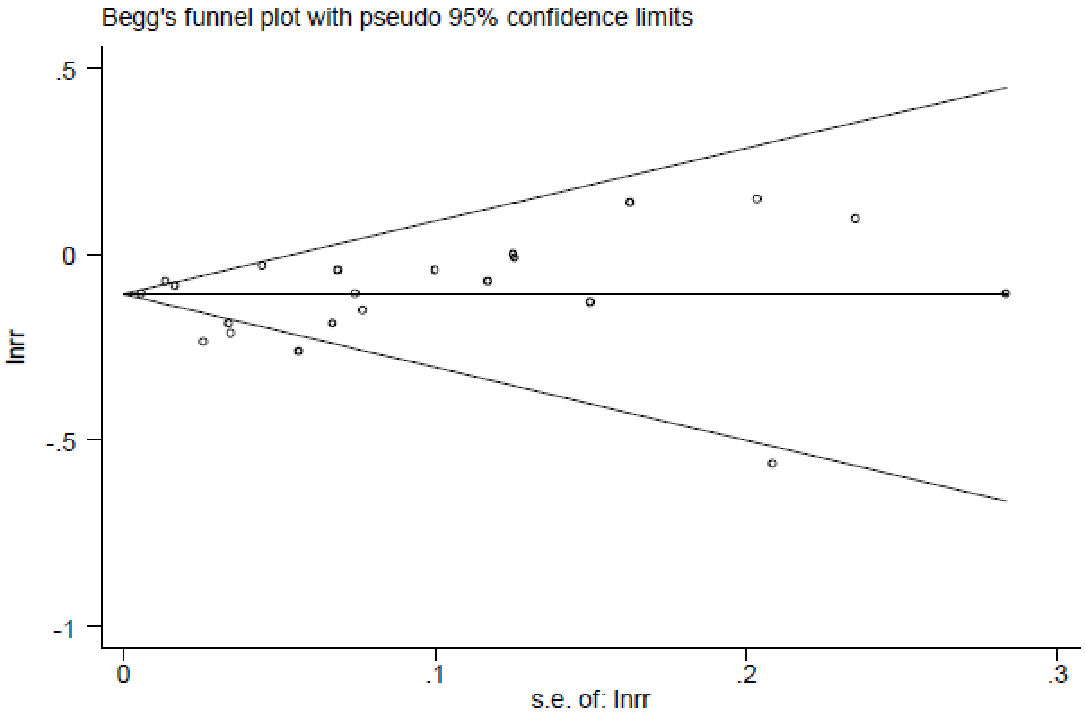

Supplementary Figure S2. Begg's funnel plot examining potential publication bias.
